# Therapeutic Potential of AAV1-Rheb(S16H) Transduction Against Alzheimer’s Disease

**DOI:** 10.3390/jcm8122053

**Published:** 2019-11-22

**Authors:** Gyeong Joon Moon, Sehwan Kim, Min-Tae Jeon, Kea Joo Lee, Il-Sung Jang, Michiko Nakamura, Sang Ryong Kim

**Affiliations:** 1 School of Life Sciences, Kyungpook National University, Daegu 41566, Korea; neobios7@gmail.com (G.J.M.); arputa@naver.com (S.K.); mirinae109@nate.com (M.-T.J.); 2 BK21 plus KNU Creative BioResearch Group, Kyungpook National University, Daegu 41566, Korea; 3 Neural Circuits Research Group, Korea Brain Research Institute, Daegu 41062, Korea; relaylee@kbri.re.kr; 4 Brain Science and Engineering Institute, Kyungpook National University, Daegu 41566, Korea; jis7619@knu.ac.kr; 5 Department of Pharmacology, School of Dentistry, Kyungpook National University, Daegu 41940, Korea; michiko21a@hotmail.com; 6 Institute of Life Science & Biotechnology, Kyungpook National University, Daegu 41566, Korea

**Keywords:** Alzheimer’s disease, Rheb(S16H), neurotrophic signaling, β-amyloid, cognitive impairment

## Abstract

We recently reported that adeno-associated virus serotype 1-constitutively active Ras homolog enriched in brain [AAV1-Rheb(S16H)] transduction of hippocampal neurons could induce neuron-astroglia interactions in the rat hippocampus in vivo, resulting in neuroprotection. However, it remains uncertain whether AAV1-Rheb(S16H) transduction induces neurotrophic effects and preserves the cognitive memory in an animal model of Alzheimer’s disease (AD) with characteristic phenotypic features, such as β-amyloid (Aβ) accumulation and cognitive impairments. To assess the therapeutic potential of Rheb(S16H) in AD, we have examined the beneficial effects of AAV1-Rheb(S16H) administration in the 5XFAD mouse model. Rheb(S16H) transduction of hippocampal neurons in the 5XFAD mice increased the levels of neurotrophic signaling molecules, including brain-derived neurotrophic factor (BDNF) and ciliary neurotrophic factor (CNTF), and their corresponding receptors, tropomyosin receptor kinase B (TrkB) and CNTF receptor α subunit (CNTFRα), respectively. In addition, Rheb(S16H) transduction inhibited Aβ production and accumulation in the hippocampus of 5XFAD mice and protected the decline of long-term potentiation (LTP), resulting in the prevention of cognitive impairments, which was demonstrated using novel object recognition testing. These results indicate that Rheb(S16H) transduction of hippocampal neurons may have therapeutic potential in AD by inhibiting Aβ accumulation and preserving LTP associated with cognitive memory.

## 1. Introduction

Alzheimer’s disease (AD), the most prevalent progressive neurological disorder, causes the decline of cognitive function and memory loss; currently, only symptomatic treatments are available [1,2]. Despite progress in elucidating the underlying mechanisms and genes involved in certain hallmarks of AD, including neuronal degeneration, extracellular neuritic plaques, and intracellular neurofibrillary tangles, the mechanisms underlying the degeneration and functional disruption that occur in AD remain unknown, and treatments affecting progression of the disease are limited [1,3,4]. This incomplete understanding of the etiology of AD hinders the development of knowledge-based targeted therapeutics. Many studies have suggested that alterations in the levels of specific neurotrophic factors (NTFs), such as brain-derived neurotrophic factor (BDNF) and ciliary neurotrophic factor (CNTF), are associated with the pathogenesis of neurodegenerative diseases such as AD and Parkinson’s disease (PD) [3,5,6,7,8,9,10,11]. Others have suggested that upregulation of NTFs mitigates neuronal death and enhances neuronal function and plasticity in AD and PD [12,13,14].

We have previously reported that the upregulation of NTFs by activation of a mammalian target of rapamycin complex 1 (mTORC1) following adeno-associated virus 1 (AAV1) transduction with a gene encoding a constitutively active human Ras homolog enriched in brain [Rheb(S16H)] protects against neurodegeneration in animal models [9,15,16,17]. Also, we observed that Rheb(S16H) transduction protects neurons from thrombin-induced neurotoxicity through neuron–glia interactions in rat hippocampus [18]. Others have reported that Rheb is dysregulated in the brains of patients with AD [19], and that the control of Rheb expression promotes the accumulation of β-site amyloid precursor protein-cleaving enzyme 1 (BACE1) in the adult brain [19] and elicits spatial memory deficits in vivo [20]. Taken together, these results suggest that Rheb may be an important regulator for neuronal survival and cognitive memory in the adult brain. However, it remains uncertain whether Rheb(S16H) transduction of hippocampal neurons could induce beneficial neurotrophic effects and preserve cognitive memory in an animal model of AD with characteristic phenotypic features, such as β-amyloid (Aβ) accumulation and cognitive impairments. In the present study, therefore, we examined whether adeno-associated virus serotype 1-constitutively active Ras homolog enriched in brain [AAV1-Rheb(S16H)] administration induces beneficial effects in 5XFAD mouse model, a mouse model of amyloid deposition that expresses five familial AD (FAD) mutations and recapitulates major features of AD [21].

## 2. Materials and Methods

### 2.1. Animals

**Figure 1:**
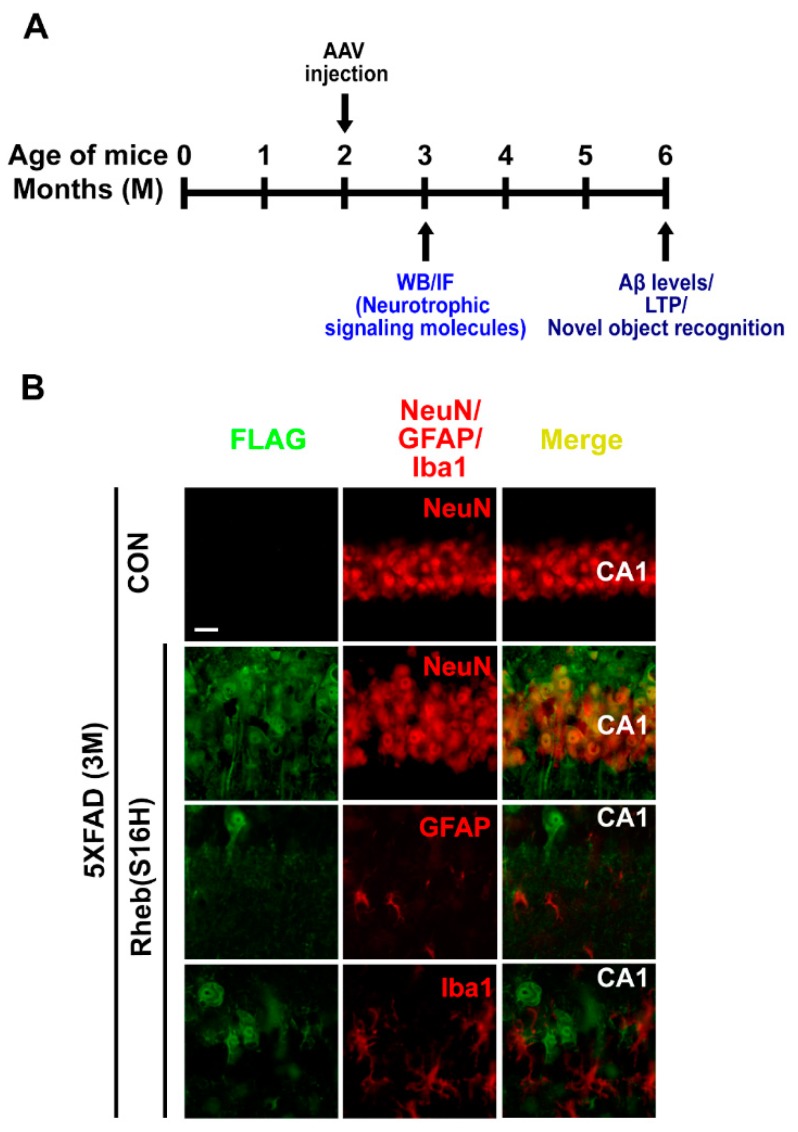
Experimental schematic and transduction of 5XFAD mouse hippocampal neurons with AAV1-Rheb(S16H). (**A**) Experimental schematic for the study of AAV1-Rheb(S16H) effects in the hippocampus of 5XFAD mice. (**B**) AAV1-Rheb(S16H) was injected unilaterally into the 5XFAD mouse hippocampus. Four weeks later, double immunofluorescence staining was performed to visualize co-expression patterns of FLAG (green) and NeuN (red), FLAG (green) and GFAP (red), or FLAG (green) and Iba1 (red). Scale bar, 20 μm.

5XFAD mice (8 weeks old, male) with a C57BL/6 background were kindly provided by Dr. Kea Joo Lee (Korea Brain Research Institute, Korea). All animal experimental procedures were conducted in accordance with the Guidelines for Animal Care and Use of Kyungpook National University, approved by the Animal Care and Use Committee of Kyungpook National University (No. KNU 2016-0042 and 2019-0002). A total of 56 mice were used in this study: 3-month-old wild-type control (WT) (n = 4) and 5XFAD (n = 14) mice, and 6-month-old WT (n = 12) and 5XFAD (n = 26) mice. Three to six mice per group were used for the long-term potentiation (LTP) experiment, and three or four 5XFAD mice per group were used for all other experiments. The precise numbers of animals are provided in the figure legends. The experimental scheme is provided in Figure 1A.

### 2.2. Production of AAV Viral Vectors

All vectors used were AAV1 serotype as previously described [15,16]. A plasmid carrying Rheb was purchased from OriGene Technologies (Rockville, MD, USA). *Rheb* DNA was amplified and modified to incorporate a FLAG-encoding sequence at the 3′-end by expanded long-template PCR (Roche, Basel, Switzerland). Constitutively active Rheb [Rheb(S16H)] was generated with a Phusion Site-directed Mutagenesis Kit (New England Biolabs, Ipswich, MA, USA) in pGEM-T vector (Promega, Madison, WI, USA) and then cloned into an AAV packaging construct that utilizes the chicken β-actin promoter and contains a 3′ WPRE (pBL). The AAVs were produced by the University of North Carolina Vector Core, and the genomic titer of Rheb(S16H) was 2 × 10^12^ viral genomes/mL. Green fluorescent protein (GFP), used as a control, was subcloned into the same viral backbone, and viral stocks were produced at titers of 1 × 10^12^ viral genomes/mL.

### 2.3. Intrahippocampal Injection

Animals were anesthetized with 360 mg/kg chloral hydrate (Sigma, St. Louis, MO, USA) by intraperitoneal (i.p.) injection and placed in a stereotaxic frame (David Kopf Instrument, Tujunga, CA, USA). AAV1-GFP or AAV1-Rheb(S16H) was infused bilaterally into the hippocampal CA1 area of 5XFAD mice (AP: −2.0 mm; ML: ±1.2 mm; DV: −1.5 mm, relative to the bregma), according to the brain atlas [22] using 30-gauge injection needles connected to a 10-μl Hamilton syringe. With an automated syringe pump, 2.0 μL viral vector suspension was infused at a rate of 0.1 μl/min over 20 min, and the injection needle left in situ for an additional 5 min to allow diffusion into the tissue and minimize dragging back along the injection track.

### 2.4. Electrophysiology

Wild-type and 5XFAD mice were decapitated under ketamine anesthesia (50 mg/kg, i.p.). The brains were quickly removed and immersed in ice-cold Ringer’s solution composed of (mM): 119 NaCl, 2.5 KCl, 1.3 MgSO_4_, 2.5 CaCl_2_, 1.0 NaH_2_PO_4_, 26.2 NaHCO_3_, and 11 glucose, and saturated with 95% O_2_ and 5% CO_2_. The hippocampi were dissected and transverse slices (400-μm-thick) were prepared with a vibrating blade microtome (VT1000S; Leica, Wetzlar, Germany). The slices were placed in a humidified holding chamber for at least 1 h and then transferred to a recording chamber that was continuously superfused at a rate of approximately 2 mL/min with Ringer’s solution. Field excitatory postsynaptic potentials (fEPSPs) were recorded at the stratum radiatum of the CA1 region using a glass microelectrode filled with 2 M NaCl in the presence of 100 μM picrotoxin, a GABA_A_ receptor antagonist. Electrical stimuli were delivered at 0.1 Hz through a bipolar tungsten electrode, and LTP was induced using theta-burst stimulation (TBS) protocols: bursts of 4 pulses at 100 Hz, with the bursts repeated in 10 trains at 5 Hz, and the trains repeated 4 times every 10 s. All recordings were done at 24–26 °C.

The electrical measurements were performed using an Axopatch-1D amplifier (Molecular Devices, Sunnyvale, CA, USA). The signal was filtered at 1 kHz, digitized at 10 kHz, and stored on a personal computer equipped with pCLAMP 10.3 software (Molecular Devices). The amplitudes of fEPSPs were calculated by subtracting the baseline from the peak amplitude. The extent of LTP was determined as the percent increase in the mean of fEPSP amplitude 50–60 min after TBS of the baseline (mean fEPSP amplitude 10 min before LTP induction).

### 2.5. Novel Object Recognition Test

Novel object recognition tests were performed as previously described [23,24], with some modifications. Before the behavior test, the mice were habituated to the open testing arena (40 × 40 × 40 cm white opaque acrylic open field) for three consecutive days (10 min each time). The arena was cleaned between trials with 70% ethyl alcohol. The tests were performed under a low illumination light in the dark for stress minimization. For the object recognition test, the mice were exposed for 5 min to one familiar object and one novel object (different shape and color). With a video camera, the object exploration time was recorded when the mice directly or indirectly touched the object with the nose, mouth, or forepaws. Mice were perceived as “interacting” with the object when the nose was in contact with the object or directed at the object within a minimal defined distance (most commonly ≤2 cm). However, contacts such as backing into the object or bumping the object in passing were excluded, as were any contacts occurring when the mouse was standing or leaning on the object to explore other aspects of the chamber.

### 2.6. Immunofluorescence Staining Procedures

Animals were transcardially perfused, and fixed brain sections (30-μm-thick) were processed for immunofluorescence staining as previously described [15], with some modifications. Briefly, brain sections were rinsed in PBS and then incubated at 4 °C for 48 h with one of the following pairs: rabbit anti-FLAG (1:3000; Sigma) and mouse anti-neuronal nuclei (NeuN, 1:500; Millipore), rabbit anti-FLAG (1:3000; Sigma) and mouse anti-glial fibrillary acidic protein (GFAP, 1:2000, Millipore), mouse anti-FLAG (1:2000; Sigma) and rabbit anti-ionized calcium-binding adapter molecule 1 (Iba1, 1:2000; Wako Pure Chemical Industries), mouse anti-NeuN (1:500; Millipore) and rabbit anti-BDNF (1:200; Santa Cruz), rabbit anti-GFAP (1:2000, Millipore) and goat anti-tropomyosin receptor kinase B (TrkB, 1:100 R&D Systems), mouse anti-GFAP (1:2000; Millipore) and goat anti-CNTF (1:100, R&D Systems), and mouse anti-NeuN (1:500; Millipore) and goat anti-CNTF receptor α subunit (CNTFRα, 1:100, R&D Systems). The sections were then rinsed with PBS-0.5% bovine serum albumin and incubated with Texas Red-conjugated anti-rabbit IgG, anti-goat IgG, or anti-mouse IgG (1:400; Vector Laboratories), and fluorescein isothiocyanate-conjugated anti-rabbit IgG or anti-mouse IgG (1:200; Vector Laboratories), for 1 h, and then washed and mounted with Vectashield mounting medium (Vector Laboratories). The stained sections were imaged by confocal microscopy (LSM700, Carl Zeiss).

### 2.7. Thioflavin S Staining

The sections were washed with distilled water and incubated with thioflavin S solution (1% w/v, Sigma) for 10 min, and then incubated with 70% ethanol for 5 min and washed with distilled water.

### 2.8. Western Blot Analysis

Western blot analysis was performed as described previously [15,16]. Briefly, the lysates obtained from astrocyte culture and hippocampal tissue were homogenized and centrifuged at 4 °C for 15 min at 14,000 × *g*. The supernatant was transferred to a fresh tube, and the concentration was determined using a bicinchoninic acid assay kit (BCA assay, Bio-Rad Laboratories, Hercules, CA, USA). The samples were boiled at 100 °C for 5 min before gel loading, and equal amounts of protein (20 μg) were loaded into each lane with loading buffer. Proteins separated by gel electrophoresis were transferred to polyvinylidene difluoride membranes (Millipore) using an electrophoretic transfer system (Bio-Rad Laboratories), and then the membranes were incubated overnight at 4 °C with specific primary antibodies. The following primary antibodies were used: mouse anti-β-actin (1:1000; Santa Cruz), rabbit anti-BDNF (1:500; Santa Cruz), goat anti-CNTF (1:1000, R&D Systems), goat anti-TrkB (1:1000, R&D Systems), goat anti-CNTFRα (1:1000, R&D Systems), rabbit anti-phosphorylated eukaryotic initiation factor 4E-binding protein 1 (p-4E-BP1, 1:1000; Cell Signaling), rabbit anti-4E-BP1 (1:1000; Cell Signaling), and rabbit anti-β-amyloid (Aβ, 1:1000; Cell Signaling). After washing, the membranes were incubated with horseradish peroxidase-conjugated secondary antibodies (Santa Cruz) for 1 h at room temperature, and the blots were developed using enhanced chemiluminescence Western blot detection reagents (GE Healthcare Life Sciences, Little Chalfont, UK). The signal was analyzed with a LAS-500 image analyzer (GE Healthcare Life Sciences) and Multi Gauge version 3.0 (Fuji Photo Film, Tokyo, Japan). All histograms show quantitative analysis based on the density of target proteins normalized to the β-actin band for each sample.

### 2.9. Statistical Analysis

All values are expressed as the mean ± standard error of the mean (SEM). Data normality was assessed by Shapiro–Wilk test, and subsequent analysis was performed by Student’s unpaired *t*-test, Mann–Whitney rank sum test, Kruskal–Wallis test, or one-way ANOVA with Tukey’s post-hoc test. All statistical analyses were performed with Sigmaplot software (Version 12.0, Systat Software, San Jose, CA, USA).

## 3. Results

### 3.1. Rheb(S16H) Transduction of Hippocampal Neurons Induced a Neuroprotective System in 5XFAD Mice

We previously reported that neuronal transduction with AAV1-Rheb(S16H) induces the production of BDNF in hippocampal neurons [16] and that CNTF production through astroglial activation following the increase in BDNF has direct neuroprotective effects and contributes to Rheb(S16H)-induced protection against thrombin-induced neurotoxicity in the rat hippocampus [18]. In this study, using a 5XFAD mouse model, we investigated the effects of intrahippocampal AAV1-Rheb(S16H) administration on neurotrophic signaling activation by (1) measuring the levels of p-4E-BP1 (indicating mTORC1 activity), BDNF, TrkB, CNTF, and CNTFRα; and (2) evaluating indicators of functional protection; namely, the preservation of LTP, which is a molecular event that contributes to learning and memory [25,26], and novel object recognition [21,27]. As reported in the previous study of hippocampal Rheb(S16H) expression [16,18], hippocampal Rheb(S16H) expression was increased in the neurons but not in the glial cells of 5XFAD mice, as demonstrated by double immunofluorescence staining (NeuN for neurons and GFAP and Iba1 for astrocytes and microglia, respectively) at 4 weeks after intrahippocampal injection (Figure 1B). To assess the effects of Rheb(S16H) expression on p-4E-BP1, BDNF, TrkB, CNTF, and CNTFRα production, 5XFAD mice were administered an intrahippocampal injection of AAV1-Rheb(S16H) at 2 months, and protein levels in the hippocampus were measured by Western blotting and double immunofluorescence staining 4 weeks after viral injection (Figure 1A).

**Figure 2:**
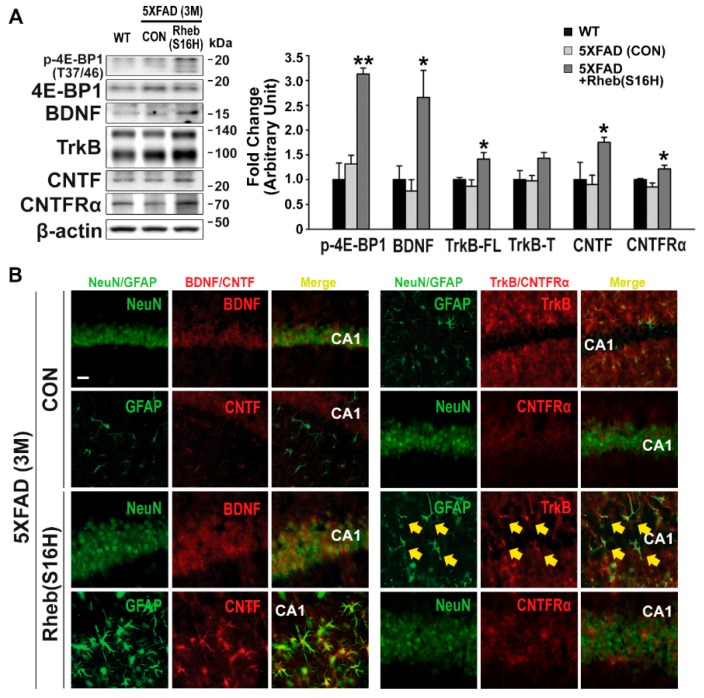
Construction of a neuroprotective system by AAV1-Rheb(S16H) transduction in the 5XFAD mouse hippocampus. Hippocampal tissue sections and protein lysates obtained from WT, untreated 5XFAD (CON), and AAV1-Rheb(S16H)-treated 5XFAD mice. (**A**) Representative bands on Western blot analysis of mTORC1 activity (p-4E-BP-1 and 4E-BP-1) and levels of neurotrophic factors [brain-derived neurotrophic factor (BDNF) and ciliary neurotrophic factor (CNTF))and their corresponding receptors [tropomyosin receptor kinase B (TrkB) and CNTF receptor α subunit (CNTFRα)) in the hippocampus of WT, untreated 5XFAD (CON), and AAV1-Rheb(S16H)-treated 5XFAD mice. Differences among groups were evaluated with the Kruskal–Wallis test or one-way ANOVA and Tukey’s post-hoc analysis. * *p* < 0.05 and ** *p* < 0.001 vs. untreated 5XFAD (CON) mice (n = 4). (**B**) Expression and localization of trophic factor signaling molecules in the hippocampus of untreated 5XFAD (CON) and AAV1-Rheb(S16H)-treated 5XFAD mice with double-immunofluorescence staining. Double immunofluorescence staining in the CA1 region of the hippocampus shows BDNF (red) and CNTFRα (red) co-localization with NeuN-positive neurons (green) and CNTF (red) and TrkB (red) co-localization with GFAP-positive astrocytes (green). Areas of co-localization are marked with a yellow arrow. Scale bar, 20 μm.

Western blots showed no significant difference in the protein levels in WT and 5XFAD mice (Figure 2A). However, similar to its effects in the rat hippocampus [18], Rheb(S16H) transduction of hippocampal neurons significantly increased p-4E-BP1, BDNF, full-length TrkB, CNTF, and CNTFRα in 5XFAD mice compared to the levels in untreated 5XFAD mice (Figure 2A; * *p* < 0.05 and ** *p* < 0.001 vs. untreated 5XFAD, respectively). Double immunofluorescence staining also showed increased BDNF and CNTFRα in hippocampal neurons and increased TrkB and CNTF in reactive astrocytes following Rheb(S16H) transduction (Figure 2B). Moreover, Rheb(S16H) transduction induced sustained increases in p-4E-BP1, BDNF, CNTF, and CNTFRα in the 5XFAD mouse hippocampus at 4 months after viral injection (Figure S1), indicating the construction of a neuroprotective system through neuron–astrocyte interactions.

### 3.2. Intrahippocampal Administration of AAV1-Rheb(S16H) Inhibited Aβ Oligomerization and Deposition in 5XFAD Mice

**Figure 3:**
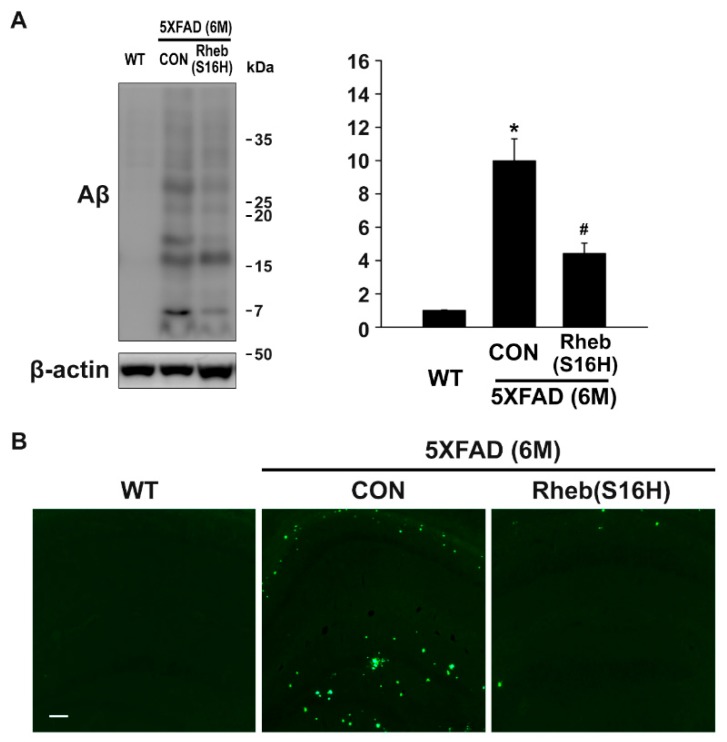
Inhibition of Aβ accumulation in the AAV1-Rheb(S16H)-treated 5XFAD mouse hippocampus. (**A**) Representative bands by Western blotting for Aβ. (**B**) Differences among groups were evaluated with the one-way ANOVA and Tukey’s post-hoc analysis at a level of significance of * *p* < 0.001 vs. WT control, and ^#^ *p* < 0.01 vs. untreated 5XFAD (CON) mice (n = 3). (**C**) Thioflavin S staining in the hippocampus of untreated and AAV1-Rheb(S16H)-treated 5XFAD mice. Scale bar, 100 μm.

To assess the effect of Rheb(S16H) transduction on Aβ aggregation, we measured Aβ accumulation and plaque burden in the 6-month-old 5XFAD mouse hippocampus using Western blots and thioflavin S staining, respectively. Western blots showed an increase in Aβ oligomerization and deposition, and these increases were significantly inhibited by Rheb(S16H) transduction (Figure 3A; * *p* < 0.001 vs. WT control, and ^#^ *p* < 0.01 vs. untreated 5XFAD mice). Similarly, thioflavin S staining showed a significantly decreased hippocampal Aβ plaque burden in the AAV1-Rheb(S16H)-injected 5XFAD mice compared to untreated 5XFAD mice (Figure 3B).

### 3.3. Intrahippocampal Administration of AAV1-Rheb(S16H) Preserved LTP and Cognitive Memory in 5XFAD Mice

**Figure 4:**
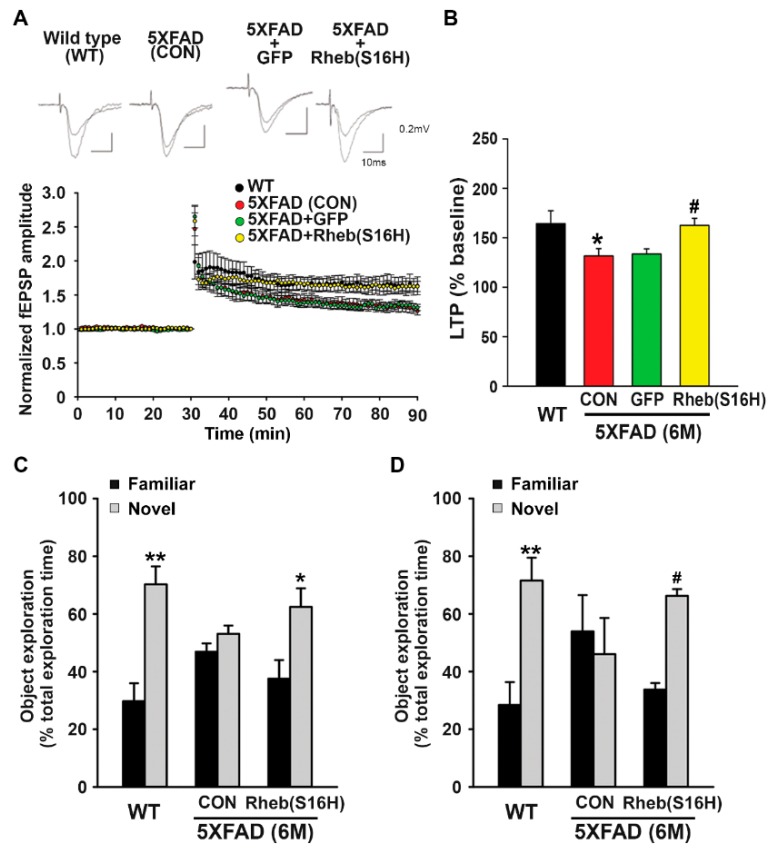
Preservation of long-term potentiation (LTP) and cognitive memory in the AAV1-Rheb(S16H)-treated 5XFAD mice. (**A**) Time courses of fEPSP responses before and after TBS from hippocampal slices in WT (black circle, n = 8 from 6 mice), untreated 5XFAD (CON) (red circle, n = 10 from 5 mice), 5XFAD-AAV1-GFP (green circle, n = 10 from 3 mice), and 5XFAD-AAV1-Rheb(S16H) groups (yellow circle, n = 6 from 6 mice). The values were normalized in each experiment to the mean amplitude measured during the control period (10 min before TBS stimulation). LTP induction was applied at 30 min. (**B**) The mean fEPSP amplitude 50–60 min after TBS of the baseline (mean fEPSP amplitude 10 min before LTP induction). Differences among groups were evaluated with the one-way ANOVA and Tukey’s post-hoc analysis. * *p* < 0.05 vs. WT and ^#^ *p* < 0.01 vs. untreated 5XFAD (CON) mice. (**C**,**D**) Novel object recognition test of cognitive impairment in animal models. Results are shown as the mean object exploration time for the short-term (**C**) and long-term (**D**) latency tests (n = 4). Data are expressed as a percentage of total time (mean ± SEM). Differences between groups were evaluated with the Student’s unpaired *t*-test or Mann–Whitney rank sum test. * *p* < 0.05, ** *p* < 0.01, and ^#^ *p* < 0.001 vs. familiar object.

To examine the effect of Rheb(S16H) transduction on cognitive function in 5XFAD mice, we performed LTP analysis and novel object recognition testing. TBS induced significant synaptic potentiation in hippocampal slices from 6-month-old WT mice (Figure 4A,B; 164.2% ± 13.1% of the baseline); by comparison, TBS-induced LTP was significantly impaired in the samples from 5XFAD mice (Figure 4A,B; 131.6% ± 7.4% of the baseline, * *p* < 0.05 vs. WT). These results are consistent with those of previous reports showing that 5XFAD mice exhibit a significant reduction in LTP levels at excitatory synapses in the hippocampus or cortex [28,29]. However, Rheb(S16H) transduction of hippocampal neurons significantly preserved LTP in 5XFAD mice compared to in untreated or AAV1-GFP-treated 5XFAD mice (Figure 4A,B; 162.5% ± 22.7% of the baseline, ^#^ *p* < 0.01 vs. untreated 5XFAD mice).

In the novel object recognition tests, 6-month-old WT mice spent significantly more time exploring novel objects than familiar objects, at both 1 and 24 h (Figure 4C,D; ** *p* < 0.01 vs. familiar objects), but the 6-month-old untreated 5XFAD mice spent the same amount of time exploring both objects (Figure 4C,D). Similar to the WT mice, 6-month-old 5XFAD mice injected with bilateral intrahippocampal AAV1-Rheb(S16H) at 2 months of age spent significantly more time exploring novel objects than familiar, at both 1 and 24 h (Figure 4C,D; * *p* < 0.05 and ^#^ *p* < 0.001 vs. familiar objects), suggesting that the effects of Rheb(S16H) transduction of hippocampal neurons, such as neurotrophic signaling activation, preservation of LTP, and inhibition of Aβ deposits, limited the functional loss associated with memory impairments in 5XFAD mice.

## 4. Discussion

Rheb, a member of the small GTPase superfamily, encodes a lipid-anchored cell membrane protein with five repeats of the Ras-related GTP-binding region [17,30]. Rheb synthesis is upregulated after toxic insults or by growth factors, and its expression mediates many cellular processes such as cell volume growth, cell cycle progression, neuronal axon regeneration, autophagy, nutritional deprivation, oxygen stress, and cellular energy status [30]. The effects of Rheb are mediated by the tuberous sclerosis complex (TSC)1/TSC2, and GTP-bound active Rheb can stimulate mTORC1 activation, which, in turn, enhances the activity of intracellular cell survival pathways [17,31]. The serine at position 16 of Rheb is sensitive to TSC GTPase activation, and the point mutation of serine to histidine in Rheb(S16H) results in persistence of GTP-bound Rheb in an activated state through resistance to TSC activation [16,17,31]. Consequently, upregulation of Rheb(S16H) protects against neurotoxicity through the sustained activation of mTORC1 in the adult brain [16,17,31,32].

The expression of BDNF, a neurotrophin that mediates neuronal survival and differentiation by binding to the specific receptor TrkB [16,33,34], is decreased in the hippocampus and cortex of patients with AD [10,16,35]. However, increased expression of full-length TrkB, which contains a catalytic kinase domain that can be activated [36,37], is observed in glial cells in the hippocampus of AD patients [38], suggesting the existence of a hippocampal neuroprotective system in AD patients. Our previous study demonstrated that the activation of mTORC1 induced by Rheb(S16H) transduction of hippocampal neurons causes BDNF production, which protects against thrombin-induced neurotoxicity in the rat hippocampus [16]. Another study showed that astrocytes are the primary recipient cells of neuronal BDNF in the visual system [39]. Astrocytic CNTF mediates neuronal survival, neurogenesis, and neuronal plasticity through CNTFRα binding in the central nervous system [40,41,42]. Recently, we also observed that neuronal BDNF induced by Rheb(S16H) transduction stimulates the production of astrocytic CNTF via increased astrocytic TrkB expression and that this upregulation contributes to neuroprotection in the thrombin-treated rat hippocampus [18].

Cumulative data from preclinical studies show the utility of BDNF and CNTF for conferring various neuroprotective effects led to the idea that these NTFs could be a clinically applicable therapeutic target in neurodegenerative diseases [16,18,43]. Nevertheless, the clinical utility of systemic BDNF and CNTF is limited by poor blood–brain barrier (BBB) permeability, short half-life, and off-target effects [44,45]. Therefore, viral transduction of hippocampal neurons through methods such as AAV1-Rheb(S16H) administration, resulting in the sustained expression of beneficial target genes, is a potential therapeutic approach for neurodegenerative diseases such as AD and PD [16,17,18,31,32]. Moreover, there are reports showing that Rheb is dysregulated in the brains of patients with AD [19] and that the control of Rheb expression upregulates BACE1 in the adult brain, resulting in Aβ accumulation [19] and spatial memory deficits [20]. However, there are no reports published to date showing that Rheb(S16H) transduction of hippocampal neurons could induce beneficial effects such as neurotrophic effects, inhibition of Aβ accumulation, and preservation of cognitive memory in a transgenic animal model of AD.

In the present study, we examined the effects of AAV1-Rheb(S16H) administration in 5XFAD mice, a transgenic animal model of AD [21]. Our findings accord with those of the previously reported rat hippocampus studies [16,18]. We observed that the intrahippocampal administration of AAV1-Rheb(S16H) in 5XFAD mice increases the levels of BDNF and CNTFRα in hippocampal neurons and of TrkB and CNTF in reactive astrocytes (Figure 2A,B), reflecting the induction of a hippocampal neuroprotective system through neuron–astrocyte interactions in the adult brain tissue. In addition, AAV1-Rheb(S16H) administration significantly reduced the oligomerization and deposition of Aβ in the hippocampus of 5XFAD mice compared to untreated mice (Figure 3A,B) and mitigated the decline of LTP in the 5XFAD mouse hippocampus (Figure 4A,B); both effects might contribute to the preservation of cognitive memory (Figure 4C,D).

This study has certain limitations that should be addressed in future studies. In this study, the 5XFAD mouse is used as an animal model that recapitulates the major features of the AD model. However, the 5XFAD mouse model does not have significant tau pathology such as neurofibrillary tangles (NFTs). The severity of NFTs tightly correlates with cognitive decline and neuronal degeneration in AD [46]. Both Aβ accumulation and NFTs may synergistically induce neurodegenerative processes related to AD [47].

## 5. Conclusions

Our results show that the upregulation of Rheb(S16H) in hippocampal neurons induces a neuroprotective system, attenuates Aβ production and accumulation, and inhibits the decline of LTP in the hippocampus of 5XFAD mice; further, it appears to inhibit cognitive impairments in this animal model. Therefore, we conclude that the transduction of hippocampal neurons with Rheb(S16H) may have therapeutic potential against AD, and that further investigation of the clinical safety and feasibility of intrahippocampal administration of AAV1-Rheb(S16H) is warranted.

## Supplementary Materials

The following are available online at https://www.mdpi.com/2077-0383/8/12/2053/s1, Figure S1: Conservation of a neuroprotective system by AAV1-Rheb(S16H) transduction in the 5XFAD mouse hippocampus at 4 months post-injection.

## Abbreviations

Rheb(S16H): Active form of ras homolog enriched in brain; AD: Alzheimer’s disease; Aβ: β-amyloid; AAV1: Adeno-associated virus serotype 1; FAD: Familial AD; BDNF: Brain-derived neurotrophic factor; CNTF: Ciliary neurotrophic factor; TrkB: Tropomyosin receptor kinase B; CNTFRα: CNTF receptor α subunit; LTP: Long-term potentiation; NTFs: Neurotrophic factors; PD: Parkinson’s disease; mTORC1: Mammalian target of rapamycin complex 1; BACE1: β-site amyloid precursor protein-cleaving enzyme 1; GFP: Green fluorescent protein; i.p.: Intraperitoneal; fEPSPs: Field excitatory postsynaptic potentials; TBS: Theta-burst stimulation; NeuN: Neuronal nuclei; GFAP: Glial fibrillary acidic protein; Iba1: Ionized calcium-binding adapter molecule 1; (p-)4E-BP1: (phosphorylated) Eukaryotic initiation factor 4E-binding protein 1; WT: Wild type; TSC: Tuberous sclerosis complex; BBB: Blood–brain barrier; NFTs: Neurofibrillary tangles.
