# Supplementary material for: Therapeutic Potential of AAV1-Rheb(S16H) Transduction Against Alzheimer’s Disease
Source: J Clin Med. 2019 Nov 22;8(12):2053. doi: 10.3390/jcm8122053 (PMC6947419; doi:10.3390/jcm8122053)
Supplement: Supplementary file 1 [file jcm-08-02053-s001.pdf]

Supplementary Figure

Therapeutic potential of AAV1-Rheb(S16H) transduction against Alzheimer's disease

Running Title: Therapeutic effects of Rheb(S16H) in 5XFAD mouse

Moon et al.

Supplementary Figure: 1

Supplementary Figure

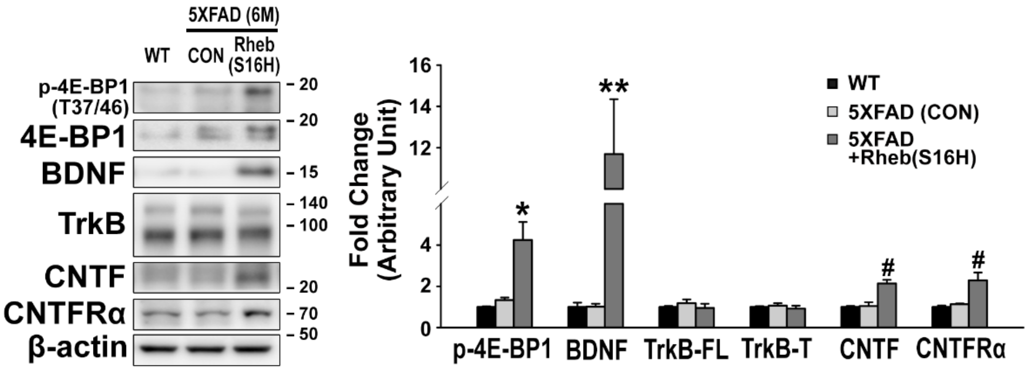

Figure S1. Conservation of a neuroprotective system by AAV1-Rheb(S16H) transduction in the 5XFAD mouse hippocampus at 4 months post-injection

AAV1-Rheb(S16H) transduction induced sustained increases in p-4E-BP1, BDNF, CNTF, and CNTFRα in the 5XFAD mouse hippocampus at 4 months after viral injection. Representative bands on western blot analysis of mTORC1 activity (p-4E-BP-1 and 4E-BP-1) and levels of neurotrophic factors (BDNF and CNTF) and their corresponding receptors (TrkB and CNTFRα) in the hippocampus of WT, untreated 5XFAD (CON), and AAV1-Rheb(S16H)-treated 5XFAD mice. Differences among groups were evaluated with the Kruskal-Wallis test or one-way ANOVA and Tukey's *post-hoc* analysis. *$p < 0.05$ and **$p < 0.001$ *vs*. untreated 5XFAD mice (n = 4).
